# Inflammatory Biomarkers in Addictive Disorders

**DOI:** 10.3390/biom11121824

**Published:** 2021-12-03

**Authors:** Alvaro Morcuende, Francisco Navarrete, Elena Nieto, Jorge Manzanares, Teresa Femenía

**Affiliations:** 1 Instituto de Neurociencias, Universidad Miguel Hernández-CSIC, Avda. de Ramón y Cajal s/n, San Juan de Alicante, 03550 Alicante, Spain; amorcuende@umh.es (A.M.); fnavarrete@umh.es (F.N.); enieto@umh.es (E.N.); jmanzanares@umh.es (J.M.); 2 Red Temática de Investigación Cooperativa en Salud (RETICS), Red de Trastornos Adictivos, Instituto de Salud Carlos III, MICINN and FEDER, 28029 Madrid, Spain

**Keywords:** inflammation, addiction, cannabinoid, alcohol, opioid, biomarker, diagnostic

## Abstract

Substance use disorders are a group of diseases that are associated with social, professional, and family impairment and that represent a high socio-economic impact on the health systems of countries around the world. These disorders present a very complex diagnosis and treatment regimen due to the lack of suitable biomarkers supporting the correct diagnosis and classification and the difficulty of selecting effective therapies. Over the last few years, several studies have pointed out that these addictive disorders are associated with systemic and central nervous system inflammation, which could play a relevant role in the onset and progression of these diseases. Therefore, identifying different immune system components as biomarkers of such addictive disorders could be a crucial step to promote appropriate diagnosis and treatment. Thus, this work aims to provide an overview of the immune system alterations that may be biomarkers of various addictive disorders.

## 1. Introduction

Inflammation is a physiological process that helps to repair tissue damage and resolve infections. However, abnormal responses or chronic inflammation can be pathological. There is an increase of proinflammatory cytokines and C-reactive protein (CRP) during inflammation, an acute-phase protein of hepatic origin that is released to the plasma in response to inflammation. These inflammatory mediators are associated with several psychiatric disorders [1,2] and have been shown to contribute to the development of neuroinflammation [3]. Neuroinflammatory processes are widely implicated in many psychiatric diseases, including major depression, anxiety disorders, schizophrenia, or substance use disorder (SUD) [4,5,6]. Peripheral inflammatory states can contribute to neuroinflammation through several alterations in various organs and tissues. Among the most notable alterations is the disruption of the blood–brain barrier, which facilitates the infiltration of inflammatory mediators into the central nervous system and alterations in the intestinal permeability that are associated with changes in the composition of the gut microbiota. Consequently, an increase in microbiota products and derivatives [7] may play a relevant role in activating the innate immune system, triggering a systemic inflammatory reaction followed by an adaptive immune response [8].

One of the main routes of starting the innate immune system is through the nuclear factor kappa-light-chain-enhancer of the activated B cell (NF-kB) signaling pathway. This pathway promotes the release of proinflammatory cytokines such as Interleukin-1 beta (IL-1β) and tumor necrosis factor-alpha (TNF-α), influencing the neurobiological processes that occur during the development of addiction [9]. Cytokines are small proteins that have a specific effect on the interactions and communications between cells. Some of them, such as IL-6 or IL-10 and INF-y, may carry mixed actions with pro- or anti-inflammatory actions depending on the specific situation [10,11]. In turn, proinflammatory cytokines can cross the Blood Brain Barrier (BBB) to enter the brain and to trigger neuroinflammation in addition to prompting BBB disruption. CRP also contributes to the leakage of the BBB [3] and has been positioned as a potential biomarker of neuroinflammation in major psychiatric disorders [12,13].

In addition to inflammatory cytokines, different elements are released into the extracellular space when damage takes place and are recognized by the innate immune system components. These elements are called “Damage associated Molecular Patterns” (DAMPS) and are recognized by specific receptors, which are known as Pattern Recognition Receptors (PRR), which are composed of several families of receptors. Within the PRRs, Toll-Like Receptors (TLRs) or NOD-Like Receptors (NLRs) can be highlighted. These receptors recognize DAMPS and Pathogen-Associated Molecular Patterns (PAMPS) or products that are associated with oxidative stress, such as reactive oxygen species (ROS). Once these PRRs are activated, a signaling cascade is initiated that releases inflammatory mediators through pathways such as the NF-kB [14,15,16].

Recently, several studies have focused on determining the association between inflammation and SUD, which has given rise to many findings in this regard. However, it is still unclear how the immune system and inflammatory mediators may be associated with the vulnerability for the development of SUD, its progression, and whether immunotherapy may be an appropriate approach to tackle these disorders. In addition, the existence of disease-associated microglia (DAM) and disease-associated astrocytes (DAA) has been recently identified. Recent studies show how microglia and astrocytes acquire different phenotypes in neuroinflammatory and degenerative diseases and aging [17,18,19,20]. As a result of a complex process, their phenotype can be protective or associated with the disease. Immune cells that do not release high proinflammatory cytokines when exposed to a trigger such as a lipopolysaccharide (LPS) cannot just be considered more anti-inflammatory. Therefore, prolonged neuroinflammation could result in a dystrophic phenotype and a non-responsive state by the microglia and astrocytes, resulting in different responses in the release of proinflammatory cytokines. In the case of substance use disorders (SUD), studies related to DAM and DAA and the specific phenotypes associated with the development and the stage of the disease are still very limited.

It has been shown how different cell types can synthesize proinflammatory cytokines, both neuronal and glial, and how they can substantially affect brain physiology, such as IL-6 or IL-1β in Long Term Potentiation (LTP), memory, and synaptic plasticity [21,22]. These neuroadaptive changes also occur in SUD, where alterations in the innate immune system associated with areas of the mesolimbic system and processes involved in addictive behaviors have been seen [9,23,24]. On the other hand, among TLRs, of which 13 have been described in mice and only 10 have been described in humans [16], the most studied receptor was TLR4. This receptor is involved in behavioral deficits that result due to alcohol consumption [25], alterations in synaptic plasticity, and opioid drug reinforcement [26]. However, the growing interest in the innate immune system highlights the involvement of other receptors, such as TLR3, TLR7, and NPRL3, in the case of substance disorders that are related to alcohol [27,28,29]. Furthermore, these mechanisms are postulated to be the potential therapeutic targets for substance use disorders [24].

The diagnosis and prognosis of SUD are limited by the lack of specific biomarkers, with clinical diagnosis largely being based on addiction scales and questionnaires as the primary classification tool. This is a significant disadvantage compared to other diseases, as patients with very heterogeneous clinical features are classified under the umbrella of a single diagnosis. Therefore, to improve the therapeutic process, identifying more specific and sensitive markers, particularly biomarkers that have good reproducibility, a good signal-to-noise ratio, and, very importantly, reflect the dynamism and clinical progression of the disease, are needed [30]. The purpose of this review is to evaluate the role of the immune system in substance use disorders and focuses on substances that act as nervous system depressants to assess its potential use as a biomarker and to guide future research.

## 2. Methods

The literature review searched for scientific information in the Medline database (PubMed) and employed Medical Subject Headings (MeSH). A total of three search boxes were employed according to the total number of drugs included in the review. The terms “cannabinoid,” “alcohol,” and “opioid” were combined with the term “inflammatory markers” by the Boolean operator “AND”. All of the authors critically analyzed the results of each search to decide the selection of the references according to the adequacy of its content with the subject matter of the study. No PubMed filters were applied to maximize the selection of all of the available and appropriate information. All original articles, systematic reviews, or meta-analyses identifying relevant inflammatory biomarkers in cannabinoid, alcohol, or opioid addiction were accepted. Those articles not related to the topic of interest, not written in English, or to which access was not possible were discarded.

## 3. Cannabinoids

Cannabis, such as hashish or marijuana, is the most commonly used illicit drug worldwide. Available data suggest that the prevalence and incidence of its consumption continue to rise in the next few years, thus representing a serious public health problem [31]. Approximately 24% of patients initiating treatment for substance abuse have a cannabis use disorder (CUD) diagnosis [32]. According to the last World Drug Report [33], two hundred million people used marijuana (cannabis) in 2019. Importantly, this report also alerts about the worrying reduction in the risk perception that is associated with cannabis use among adolescents and the 4-fold increase in cannabis herb potency (the content of Delta9-Tetrahydrocannabinol (THC)). An evaluation of the global disease burden indicates that CUD encompassing intoxication, withdrawal syndrome, and dependence criteria accounted for 2 million disability-adjusted life years (DALYs) globally in 2010 [34].

### 3.1. Endocannabinoid-Mediated Regulation of the Immune System

The involvement of the endocannabinoid system in the regulation of the immune system was first described when a peripheral receptor for cannabinoids (cannabinoid 2 receptor (CB2r)) was identified and characterized in the macrophages found in the marginal zone of the spleen [35]. Human cannabinoid receptors and their gene transcripts were also identified in the blood samples from regular human volunteers who reported no prior use of marijuana [36]. Several reports using endocannabinoids, natural cannabinoids, and synthetic cannabinoids revealed a significant role of these compounds on inflammation and immunomodulation [37,38,39]. Several reports have accounted for the anti-inflammatory properties of THC and other cannabinoids through multiple immune-related mechanisms, such as the suppression of TNF-α and other cytokines such as IL-6, IFN-γ, and IL-12 [40,41,42]. Thus, accumulated evidence points to the fact that cannabis extracts could modulate immune function, an essential factor that should be considered both from the recreational and therapeutic point of view associated with the use of cannabis and its derivatives. The following sections compile the studies that have evaluated the changes that occur in different immune system targets after cannabis use, which could be useful as potential biomarkers (Table 1).

### 3.2. Consequences of Cannabis Use on Inflammatory Biomarkers

In recent years, an increasing number of animal and clinical studies have evaluated how the immune system is modified in response to exposure to cannabis or to specific cannabinoid compounds. These investigations facilitated changes in different inflammatory biomarkers, mainly cytokines and acute-phase proteins, which may provide valuable information about the immediate or long-term consequences of prolonged cannabis use on the immune system.

Among the first studies that evaluated the effect of cannabis on inflammatory parameters were those that were focused on the pulmonary system. Baldwin et al. analyzed alveolar macrophages from marijuana smokers in vitro and demonstrated lower production of TNF-α, IL-6, and granulocyte–macrophage colony-stimulating factor after LPS-induced stimulation [51]. Similarly, another study performed with airway mucosal biopsies also concluded that regular marijuana smoking was associated with significant pulmonary inflammation, particularly when combined with tobacco [57]. In addition, gene expression analyses performed in the airway epithelium of cannabis users revealed that IL-6 and several Toll-like receptors (TLR2, TLR5, TLR6, TLR9), which are involved in the initiation of the inflammatory cascade, were significantly up-regulated compared to those from the controls [52].

The effects of cannabis consumption on immune function have been evaluated in different populations of cannabis users, revealing reduced serum levels of IL-6 [53,54,55], TNF-α [48,54], and IL-2 [50]. Accordingly, animal studies also showed a significant reduction in the IL-2 and IFN-γ levels [49]. Moreover, in peritoneal macrophages that had been stimulated with LPS extracted from adolescent and adult mice (just after a 10-day THC treatment), the production of IL-1β and TNF-α was reduced while IL-10 increased. Animals who were treated during the adolescent period (10-day THC treatment) and who were evaluated during adulthood demonstrated the opposite effects on the inflammatory biomarkers [43]. Interestingly, subsequent studies following the same experimental design in mice (immediate effects of recent 10 days of THC administration during adolescence or adulthood and the long-term effects of 10 days of THC treatment during adolescence in the adulthood) evaluated changes in the levels of IL-1β, TNF-α, and IL-10 in the hypothalamus and hippocampus, revealing a very similar profile to that of the one that was reported in the periphery [44]. Thus, it appears that early life exposure to cannabis produces a long-lasting and persistent neuroinflammatory state. Indeed, another study performing a 10-day treatment with THC showed increased levels of TNF-α, inducible nitric oxide synthase (iNOS), and cyclooxygenase 2 (COX-2) and reduced IL-10 in the adult prefrontal cortex (PFC) of rats [58].

Some authors found no differences between cannabis users and the corresponding controls. For instance, the levels of IL-1β, IL-6, and TNF-α were non-significantly different between the controls and the marijuana users, and even a mild elevation of IL-6 and TNF-α was found [45]. Indeed, increased levels of total oxidant status, oxidative stress, and IL-1β, IL-6, IL-8, and TNF-α were encountered in patients with CUD [46]. In addition, the effect of cannabis use on the plasma levels of the chemokine CCL11 was explored in subjects who were presently using cannabis (current use), subjects who had not consumed cannabis for two months or more (past use), and their corresponding controls (never use). CCL11 is involved in adult neurogenesis, cognitive functions, and aging processes. In this preliminary study, current cannabis use was associated with significantly higher CCL11 levels, whereas no changes were detected between past users and control patients [59].

### 3.3. C-Reactive Protein (CRP)

Interestingly, CRP has been recently used to examine the relationship between marijuana use and systemic inflammation, including its consequences on the progression of several chronic diseases such as cardiovascular disease (CVD), though mixed literature is available. One of the first studies analyzed data from the National Health and Nutrition Examination Survey (NHANES), which indicated that lower CRP levels (<0.5 mg/dL) were found in past marijuana users compared to in current and never-users [63]. These results were intriguing since lower inflammation values were present among former cannabis smokers but not among active smokers. For that reason, more recent NHANES data were employed to determine that the current marijuana users demonstrated lower CRP levels than the never-users over one year, failing to fully replicate the previous results but suggesting an anti-inflammatory effect of marijuana [64].

Despite the anti-inflammatory properties that can be attributed to marijuana use, several authors have found no robust predictive relationship with lower CRP levels [60,65,66]. Current marijuana use was associated with lower CRP levels, gender, body mass index (BMI), and anti-inflammatory medication use [61]. Similarly, a recent report also described lower systemic inflammation biomarkers, including high-sensitivity CRP, IL-6, and fibrinogen among patients who self-reported cannabis use in the past 30 days. However, these findings were not statistically significant after they were adjusted with multivariable models [62].

Costello et al., found a proinflammatory effect of marijuana on CRP in adolescents. In addition, higher CRP levels predicted cannabis use, abuse, or dependence [67]. Indeed, maternal CRP levels appear to be a predictive factor of future cannabis use during adolescence [69]. Additional studies have also described that long-term cannabis use is associated with increased levels of CRP, suggesting a potential proinflammatory profile. A cross-sectional study with 24 long-term cannabis users detected higher circulating CRP levels, which were associated with more significant 18-kDa translocator protein (TSPO) levels in the brain, which were analyzed by positron emission tomography (PET) [47]. The mitochondrial TSPO protein is highly expressed in glial cells. It is commonly used as a biomarker for neuroinflammation imaging, as its expression is up-regulated in reactive microglia and astrocyte cells in CNS diseases. However, neuronal activity can modify TSPO levels, suggesting that non-inflammatory interpretations should be considered in some physiological or pathological contexts [70]. Interestingly, Lisano et al. found higher CRP levels in cannabis users, placing them at moderate risk for CVD development [56,68].

Continued marijuana use (i.e., past 30 days) may confer a more potent anti-inflammatory effect than more remote use (i.e., past 12 months). Future studies assessing the influence of the timing of marijuana use on CRP may be critical to informing our understanding of the length of time in which marijuana use affects CRP levels.

## 4. Alcohol Use Disorder

Alcohol consumption is a central disabling element worldwide and is a risk factor for cancer development and for increasing all-cause mortality rates [71]. There are no specific biomarkers to Diagnose Alcohol Use Disorder (AUD) or to estimate the degree of consumption. According to the “Diagnostic and Statistical Manual of Mental Disorders 5th Edition” (DSM-V) [72]. At least 2 of the 11 items listed in the manual are needed to establish the diagnosis. Similar to other substance use disorders, this latest version includes both alcohol abuse and alcohol dependence.

Regarding the risk that is involved in consumption, various strategies have been developed without much prognostic sensitivity, such as evaluating the consumption of standard units. At this point, an approximate criterion of ranges can be applied that vary, such as those demonstrating a low risk of consumption, which range between 20–40 g per day (<200 g per week) for men and between 10–30 g per day (<140 g per week) for women [73]. This criterion varies between countries and the patient’s gender, so it is not uncommon to see diverse values depending on the source that is consulted. In addition, due to the importance of burden at a global dimension, the World Health Organization has developed another tool that is widely used in clinical practice, the Alcohol Use Disorders Identification Test (AUDIT). This scale evaluates consumption, providing values for low, medium, moderate, moderate risk, high risk, and probable addiction according to the score that is obtained [74].

These assessments help predict one of the direct consequences of excessive alcohol consumption, such as the development of alcoholic liver disease, alcoholic fatty liver disease, cirrhosis, hepatitis, or alcoholic hepatic encephalopathy. However, they do not provide the information that is necessary for estimating a substance use disorder. This damage may be evaluated using serum biochemical tests. Other nonspecific AUD tests can be used to estimate and relate liver damage to excessive alcohol consumption, such as the AST: ALT ratio and elevated GGT or mean corpuscular volume (MCV) [75], together with validated tools for stratifying liver damage, such as the Child–Pugh test [76].

In conjunction with the validated tools for stratifying liver damage, alcohol consumption leads to alterations in the immune system during the different phases of liver damage and sporadic consumption, so there is a complex and dynamic relationship between inflammation and alcohol consumption. Therefore, due to the impact of alcohol consumption in society and the lack of biomarkers, we highlight a series of immune alterations that have been observed in different studies during acute or chronic drinking and withdrawal to guide the development of more specific AUD biomarkers that are based on inflammatory markers. Moreover, in this section, we also evaluate the association between alcohol liver disease and inflammation.

### 4.1. Acute Drinking

The National Institute considers binge drinking on Alcohol Abuse and Alcoholism (NIAAA) as consuming 4/5 or more alcoholic beverages in less than two hours. This type of consumption mainly occurs in adolescents and young patients. In a significant number of cases, emergency medical teams must be involved, and is an essential factor that deteriorates interpersonal relationships, which has a significant negative impact on the psychosocial development of these adolescents and young people. Therefore, occasional but excessive consumption represents a significant risk to physical and mental health [77].

As for how this consumption affects the immune system, several studies have shown that binge consumption produces endotoxemia [78,79], leading to increased intestinal permeability. Endotoxins are Pathogen-Associated Molecular Patterns (PAMPs) that can trigger an innate inflammatory response with the consequent release of proinflammatory cytokines through binding to Pattern Recognition Receptors (PRR), which is the case when the bacterial lipopolysaccharide LPS travels through Toll-Like Receptor 4 (TLR4) [80]. Furthermore, it has been seen that at the intestinal level, acute alcohol consumption can increase the *Escherichia coli*/*Lactobacilli* ratio [79], which may also increase the permeability of the intestinal barrier [81].

Cytokines such as IL-8 increase in the plasma in healthy individuals after binge drinking [82]. Furthermore, a transient rise in neutrophils or peripheral blood mononuclear cells has also been reported [83,84]. On the other hand, a decrease in microRNA, which are small fragments of about 25 nucleotides of noncoding RNA, was also found. These miRNAs can act as post-transcriptional regulators of different immune system pathways [85]. In particular, miRNA-223 decreases in alcoholics with recent excessive drinking serum [83], which is highly expressed in the neutrophils and inhibits the expression of Il-1β and IL-6 mRNA through the IKKα pathway [86]. In addition, other miRNAs, such as mir-146a-5p, mir-21-5, and mir-182-5p, are altered after acute excessive alcohol consumption and in a sex-dependent manner in females, both human and mice [87].

Interestingly, these findings indicate that acute alcohol consumption modulates the inflammatory response and that it is independent of the liver damage that is caused by chronic consumption. Overall, these alterations could be helpful as a means to measure the vulnerability of developing a chronic alcohol disorder, the probability of a relapse in alcoholic patients, and detecting binge drinking consumption (Table 2).

### 4.2. Chronic Drinking

Chronic alcohol consumption has become a significant problem in society, representing one of the most significant burdens on health and social systems [88]. Moreover, alcohol use disorder dramatically impacts the social, employment, and family life of patients who are afflicted with this disorder. AUD often occurs with more significant comorbidity of psychiatric pathology or an increased risk of metabolic diseases [89].

Therefore, it is not surprising that alcohol consumption is associated with systemic inflammation that is measurable in different organs and tissues, such as in peripheral blood. A decrease in magnesium levels was found in patients with a high level of consumption compared to those with moderate consumption [90]. Low magnesium levels are associated with cell damage and cell death and damage-associated molecular patterns or DAMPS [91], activating an immune-inflammatory response. Moreover, anti-acetaldehyde antibodies can be detected in serum [92]. This adaptive response could be produced by the peripheral dendritic cells (D.C.) that are involved in the innate response [93]. A study by Laso et al. reported how D.C.s released IL1β, IL6, IL12, and TNFα in chronic alcoholic patients without liver disease [94]; furthermore, post mortem studies revealed that alcoholic patients with alcoholic ketoacidosis had elevated C-reactive protein (CRP) and higher levels of IL-6 and IL-10 in their femoral venous blood [95]. Chronic alcohol consumption increases intestinal permeability, resulting in the subsequent entry of pathogens into the bloodstream, increasing endotoxemia, which may precede the development and progression to alcoholic liver disease [96].

Moreover, chronic alcohol consumption in animal models revealed a decrease of miR181b-3p in the liver. Importantly, miR181b-3p binds to importin α5 and regulates TLR4 pathway signaling, resulting in the increased signaling of the NF-κB inflammatory pathway [97]. Moreover, an increase in miR-155 was found at the central level [98] and was found to regulate the adaptor protein MyD88 from the NF-κB signaling pathway [99]. Several alterations in the immune system have been described in the brain, such as leukocyte infiltration and microglia activation in the cortex [29]. Similarly, in post mortem brain samples, another study reported increased IBA1 and Glut5 microglia-positive cells in the cingulate cortex, while Glut5-positive microglial cells were also found in the midbrain VTA regions [100]. Furthermore, another study showed elevated protein levels of TLR7, high-mobility group box-1 HMGB1, and CD11b in the hippocampus of alcoholic patients, highlighting the relationship between TLR7 gene expression and years of alcohol consumption [28].

Changes in the gene and protein expression levels in different brain areas were found in several neuroinflammation markers, including proinflammatory cytokines. The most studied were TNF-α, IL-1β, or MCP-1. However, other cytokines or chemokines such as CXCL10, CX3CL1, CXCL2, IL-12, IL-18, and IL-33 increased in animal models and humans [27,29,98,100,101,102], while CCL4 expression was reduced [29]. Regarding other the components of the immune system, an increase in TLR3 mRNA expression was detected 24 h after the last period of alcohol consumption in the nucleus accumbens, while it was shown to have decreased in the amygdala, together with TLR4.

Some contradictory results were found in a series of neuroimaging studies. While rat and non-human primate studies increased TSPO binding activity [103,104], human studies show a reduction [103,105,106]. These differences could be because the human studies were conducted with addicts who had experienced several years in withdrawal at different time points, starting from two days of withdrawal up to a month. Other factors such as the consumption of other drugs may have interfered with the results that were observed [107].

Altogether, these findings suggest generalized inflammation after chronic alcohol consumption in both the periphery systems and in the brain (Table 3).

### 4.3. Withdrawal

In the alcohol withdrawal phase, a decrease in proinflammatory cytokines, together with an increase of anti-inflammatory ones, was found a few days after the cessation of consumption [82]. Furthermore, in patients with Alcoholic Liver Disease, CCL18 expression was increased in the adipose tissue after one week [108]. This chemokine induces an M2 macrophage phenotype [109] that is related to an anti-inflammatory response [110]. Therefore, these findings suggest that the inflammatory effect of alcohol begins to revert rapidly when alcohol consumption stops.

These changes were also found up to one month after the cessation of alcohol consumption [111]. Consistently with previous findings, advanced glycation end products (AGE) remain elevated one month after the termination of consumption [112], and decreases in Clara Cell secretory protein (CC16) [111], a protein with anti-inflammatory properties that has been postulated as a biomarker in several lung diseases, have also been observed [113].

Therefore, improving inflammatory parameters may provide a basis for detecting relapses in patients undergoing alcohol withdrawal in the short and medium term. However, it is necessary to investigate the potential of inflammatory biomarkers during a more prolonged detoxification period and to predict the patients who are the most susceptible to delirium tremens due to alcohol withdrawal, a condition that could compromise their lives (Table 4).

### 4.4. Alcoholic Liver Disease

One of the most visible consequences of the chronic, long-term consumption of large amounts of alcohol is the progression to liver disease (ALD). The disease progresses from the initial stages with fatty liver disease, which is reversible in many cases, to liver inflammation, and finally to loss of function due to liver fibrosis or cirrhosis.

Alterations in the levels of TNF-α and HSP70 [114] were found in HSP70 from the early stages of AML. It is a well-known DAMP that is associated with low-grade inflammation. This low-grade inflammation can be measured by the High-sensitivity C-reactive protein (hsCRP), which has a lower threshold than conventional CRP. Furthermore, the hsCRP values correlate positively with the markers of hepatic dysfunction [115].

Additionally, in an animal model of binge and escalating alcohol exposure, levels of IL-6, IL-1β, and TNF-α together with IL-10 were increased [116]. At the same time, IL-10 decreased after 12 weeks, indicating an imbalance in the level of proinflammatory and anti-inflammatory cytokines that concurred with the appearance of liver fibrosis.

A study with patients positively correlated the soluble suppression values of tumorigenesis-2 (sST2) with IL-6, IL-1β, the severity of the Child–Pugh scale, and the Maddrey test score used to discriminate the severity of alcoholic hepatitis. Interestingly, this study did not show an elevation in IL-33 levels [117]; IL-33 is the ligand of ST2. It should be noted that the measurement of sST2 is postulated to be a prognostic biomarker in heart failure [118].

As for the progression of fibrosis and the already established and other types of biomarkers that could assess prognosis, an increase of YKL40, a molecule that is involved in the maturation of the monocytes to macrophages that are linked to inflammation and fibrosis development, was also found [119]. In addition, in terms of survival rates, a decrease in Treg lymphocytes and an increase in Th17 lymphocytes were observed in non-surviving patients after admission for alcoholic liver disease. In the same study, an increase in IL-17a, IL-1β, and IL-6 was found, with increased IL-6 being a diagnostic and prognostic biomarker to fatal ALD [120]. Accordingly, patients with an IL-6 level ≥ 38.66 pg/mL [121] at hospital admission had significantly elevated mortality than those with low levels. Additionally, IFN-γ with antifibrotic effects decreased in patients with higher long-term mortality in AAH [122].

Furthermore, in the liver of a mouse animal model of ALD, there was an increase of pattern recognition receptors for gene expression, such as TLR1, TLR2, TLR4, TLR6, TLR7, TLR8, and TLR9 [123]. An increase of the microglia in the subventricular zone (SVZ) and the superior frontal gyrus (SFG) and increased levels of IL-6 in the percental gyrus (PCG) [124] were found in post mortem samples of alcoholic patients with cirrhosis and hepatic encephalopathy.

On the other hand, some studies suggested that the severity of acute alcoholic hepatitis can be assessed with the sCD163 protein [125], a protein that is associated with the activation of macrophages and the titer of antibodies against *Porphyromonas gingivalis* 33,277 and w38 [126], suggesting an increase in endotoxemia.

Therefore, in liver disease, due to prolonged and heavy alcohol consumption, potential biomarkers that assess and reflect the dynamism and progression of the disease based on inflammatory markers have been observed and may be able to establish the basis for future research (Table 5).

## 5. Opioid Use Disorder

Opioid use disorder is a social warming epidemic that affects more than 16 million people and causes more than 120,000 deaths per year worldwide [127]. Diagnosis is based on the American Psychiatric Association’s DSM-5. It includes, among others, an intense craving for opioids, continued opioid use despite associated social, physical, or psychological problems, the presence of tolerance, and the presence of withdrawal. Heroin represents the classical situation opioid of abuse, but other opioids derived from medical use include morphine, codeine, fentanyl, and synthetic opioids such as tramadol and oxycodone [127].

Opioids can interfere with the immune system by participating in immune cell function and by modulating the innate and acquired immune responses [128]. One of the significant concerns related to chronic opioid use is the development of immunosuppression, thereby increasing the risk of chronic disease and infection [128]. Indeed, recent data suggest that the effect on immune function is much more complicated. Opioids act as biological response modifiers, and their actions are highly contextual, plastic, adaptable, and influenced by other processes or pathophysiological conditions. In particular, emerging growing evidence points to neuroinflammation as being crucial for addictive process [129]. To clarify the role of inflammation on opioid addiction and the potential use of inflammatory biomarkers in this disorder, we mainly focused on reports investigating the effects of opioids in chronic exposure and the context of addiction. We discarded reports that were related to the inflammation-modulatory roles of opioids in pain or analgesia and other conditions.

### 5.1. Acute Opioid Administration

Acute opioid administration is related to several immune alterations. However, its relationship to the future development of addiction and the time-course modifications that occur with prolonged administration have been sparsely investigated. Interestingly, in this context, an essential role for anti-inflammatory cytokine IL-10 has been described. Morphine administration-activated glia in the rat nucleus accumbens (NAc) is concomitant with an increase in CRP, IFN-γ, CXCL9, CCL11, CCL12, CCL25, CCL17, CCL4, CCR4, and IL-10 expression and a decrease in CX3CL1 (fraktaline) [130]. The neonatal handling and pharmacological inhibition of glia in adulthood that is induced by ibudilast significantly increased the baseline expression of the anti-inflammatory cytokine IL-10 in the NAc. This action attenuated morphine-induced glial activation and cytokine/chemokine expression and prevented future morphine addiction in adulthood after morphine exposure. This idea supports the fact that IL-10 may be precisely related to this mechanism and that it could therefore be used as a potential marker in animal studies or in post mortem brains. In line with this, it was found that IL-10 mRNA expression within the NAcc negatively correlated with increased risk of the drug-induced reinstatement of Conditioned Place Preferences (CPP), suggesting a protective role for this specific cytokine against morphine-induced glial reactivity and the drug-induced reinstatement of morphine CPP.

Moreover, the authors did not find peripheral immune activation, as levels of peripheral IL-1β were not affected, whereas IL-10 significantly increased in the periphery. However, in contrast to CNS, handled rats showed decreased peripheral IL-10 levels [130]. Similarly, acute morphine administration did not change the serum levels of IL-1β and IL-6, which was not the case after 6 days of morphine administration [131]. In contrast to chronic administration, acute morphine administration also increased IL-1β levels in several brain regions, including in the striatum and hippocampus, and TNFα was also shown to be increased in the cortex [132]. Interestingly, chronic pain is accompanied by a proinflammatory profile. Several results point out that these patients have an increased risk of developing opioid addiction [133]. Notably, basal differences in immune mediators that are related to the concomitant pathology and individuals may play an essential role in the development of addiction, which should be considered.

Overall, these data suggest that acute opioid administration has different immunomodulatory effects in the periphery and CNS, influencing future addictive behavior. However, there is still a lack of information regarding how acute opioid use and its interaction with the immune system relates to the development of addictive behavior (Table 6).

### 5.2. Chronic Opioid Administration

The chronic use of opioids produces dependence, tolerance, and addiction that are mediated by immune dysregulation (Table 7). Chronic opioid administration up-regulates peripheral IL-6, IL-1β, TNFα, CRP [131,134,135]. Moreover, opioid exposure in the prenatal period can impact the peripheral inflammatory profile after birth, as shown by increased serum levels of IL-1β, IL-6, TNFα, and CXCL1, with IL-1β continuing to be increased for over a month. Moreover, isolated peripheral blood mononuclear cells (PBMC) increased basal TNFα release as well as increased LPS-induced TNFα and IL-1B release, showing that prenatal opioid exposure can reprogram the immune system to sustained peripheral activation and reactivity [136].

Several studies demonstrated that microglial activation occurs in the context of opioid addiction in the central nervous system [149,150], and recent evidence suggests that activated glia, including astrocytes and microglia, play an essential role in opioid addiction. For example, it has been shown that astrocytes are involved in the dependence that is induced by repeated morphine treatment [151] and microglia in the NAc in the acquisition and maintenance of dependence, although it is not involved in the expression of morphine-induced reward in the CPP [152]. Consistently, glial inhibitors decrease opioid addiction and physical dependence [130,152,153]. Transcriptomics and metabolomics analysis related chronic opioid exposure with the upregulation of inflammatory pathways in the brain [154,155], further demonstrating the involvement of inflammation in opioid addiction.

The release of proinflammatory cytokines in the CNS was associated with addictive behavior. In particular, in the NAc, a significant upregulation of IL-6, TNFα, and IL-1β [131,137] and IL-6 and IL-1β in mPFC [131] were found. Moreover, TLR2 and TLR4 expression were shown to have increased after chronic opioid administration in the NAc in rodents [137,138], whereas NF-kB, TNFα, and IL-6 are up-regulated in whole-brain homogenate after chronic tramadol exposure [134]. Accordingly, other studies found that chronic morphine administration induces the expression of the chemokine CCL5 in the cortex and striatum [132] and CXCL12 in VTA, which has been related to the acquisition and maintenance of morphine-induced conditioned place preference (CPP) [139]. However, some contradictory results have also reported that chronic morphine administration did not increase the mRNA TNFα and IL-1β levels in the cortex, striatum, and hippocampus, whereas IL-1β was downregulated in the cortex [132]. Additionally, an essential role of IL-10 has been further demonstrated by Lacagnina et al. This author showed that the intracranial administration of IL-10 into the NAc reduces remifentanil self-administration by suppressing proinflammatory cytokine and chemokine gene expression [138].

Human studies also revealed several immunological alterations in the context of Opioid Use Disorder (OUD). Opioid addicts have increased plasma levels of sCD40L, TNF-α, TGF-α, IL-8, and CRP, and complement factors C3 and C4 [140,141,142,145]. Interestingly, increased levels of TNFα and IL-8 were positively correlated with years of using heroin [145].

On the other hand, total antioxidant capacity (TAC) was positively and significantly correlated to nitric oxide production in opium smokers, again suggesting a regulatory response that can occur in opium addicts to control the inflammation [141]. Additional reports have described increased inflammation in opioid addicts, together with increased white blood cell count, neutrophil count, and neutrophil percentage, while the lymphocyte percentage and basophil count were significantly lower [156]. Heroin abusers show adaptive immunity alterations, as seen by chronic B cell activation and increased total B cells [140]. Moreover, peripheral blood leukocytes (PBL) in heroin addicts showed the enhanced spontaneous production of IL-6, whereas the stimulation of PBL with con-A increased IL-10 release and proliferative activity compared to controls [143]

Identifying the inflammatory biomarkers of opioid addiction in the human brain is less advanced, but recent reports highlight the importance of immune response dysregulation. For example, a profound inflammatory profile is found in the heroin-related human post mortem brain, as shown by the increased expression of IL-15, CD68, TNF-α, IL-6, COX-2, and HSP-70, especially in the brainstem [144]. Consistently, a recent report revealed that in post mortem samples from the midbrain of opioid-induced death patients, the most dysregulated gene module was firebrick3, which was highly enriched in inflammatory and immunomodulatory genes [147]. Additionally, a large transcriptomic study in the post mortem brains of OUD patients further revealed the importance of neuroinflammation in this disorder. They focused on the dorsolateral prefrontal cortex (DLPFC) and nucleus accumbens (NAc). They found that the top-activated upstream regulators in DLPFC were TNF, IL1β, and NF-κB in the DLPFC, whereas IL1β was one of the top-activated upstream regulators in NAc. Moreover, this study revealed the direct links between neuroinflammation, risky behavior, and OUD, suggesting that neuroinflammatory pathways may ultimately drive drug-seeking behavior in OUD [146]. In addition, a recent report identified IL4 and PECAM1 as potential biomarkers for opioid use disorder [148].

### 5.3. Opioid Withdrawal

Data obtained from rodents suggest an increase in inflammatory mediators in the brain during the withdrawal period that are associated with the severity of withdrawal behavior. For example, a recent report measured gene expression in astrocytes, microglia, and neurons in the central amygdala (CeA) of control, morphine-dependent, and withdrawal rats. They found inflammatory genes, such as CXCR1, TLR2, TNF, and TNFRSF1A, to be up-regulated in the morphine group. However, chronic morphine exposure did not substantially influence the transcriptional state, but morphine withdrawal did. In particular, a significant upregulation of neuroinflammatory genes, notably TNF, occurred in the withdrawal condition in all three cell types, with astrocytes showing the most profound shift [157]. Another study found an up-regulation of inflammatory cytokines and microglia proliferation in withdrawal conditions compared to in morphine-dependent conditions. In particular, withdrawal increases IL-1β in the hippocampus and striatum and TNF-α in the striatum, reducing CCL5 in the frontal cortex. In contrast with morphine-dependent conditions, no changes were observed [132].

Interestingly, increased hippocampal levels of IL-1β and TNFα were linked to the severity of withdrawal behavior. These changes were also associated with the cortisol levels related to stress and anxiety during withdrawal [158]. Furthermore, another study consistently found that elevated hippocampal IL-1β expression was associated with the severity withdrawal behavior [159]. Moreover, morphine withdrawal induces TNFα release and neuronal TNF-receptor 1 activation, which were involved in behavioral modifications during withdrawal [160]. Additionally, the treatment with venlafaxine inhibited withdrawal behavior and inhibited the up-regulation of TNF-α, IL-1β, and IL-6 that had been induced by withdrawal. Interestingly, venlafaxine failed to affect the brain with the increased concentrations of IL-10, suggesting that withdrawal is dependent on proinflammatory cytokines [161]. The involvement of neuroinflammation arises because the glial inhibitor ibudilast inhibits withdrawal behavior [162].

In patients, data from withdrawal periods were collected in the context of methadone-maintenance therapy (MMT), where several studies have shown the association with changes in the inflammatory profile. Opioid addicts under MMT significantly increased plasma IL-1β, IL-6, and IL-8 compared to healthy subjects. Interestingly, TNFα was not increased, but both TNFα and IL-6 levels were significantly correlated with the dairy methadone dosage administered and IL-1β with the duration of methadone maintenance treatment [163]. Several studies have shown that MMT significantly reduces the plasma levels of TNF-α, CRP, IL-6, and TGF-β1 [142,164]. Additionally, MMT significantly increased cognitive abilities in opioid addicts that were associated with changes in various inflammatory markers. For instance, TNF-α levels negatively correlate with the visual memory index and IL-6 levels negatively correlate with the verbal memory index [164]. Another study consistently found that CRP and TGF-β1 decreased during MMT, although in this case, IL-1β, TNFα, and IL-6 did not change.

Interestingly, the authors found a significantly positive correlation between plasma IL-6 levels and MMT outcomes, including the severity of heroin addiction and patient adherence to MMT [165]. Another study used dextromethorphan (D.M.) as co-adjuvant therapy for MMT and found that TNFα, IL-8, and opioid dependence significantly decreased after 12 weeks of MMT-DM but not in the MMT-Placebo patients. Moreover, methadone required dose increases in the MMT-Placebo groups and dose decreases in the MMT-DM group, suggesting that patients become tolerant to methadone when it is used alone. The persistence of those cytokines may be associated with the maintained dependence and development of tolerance [145]. Accordingly, a subsequent study of the effects of D.M. found a higher reduction in TNFα but not in IL-8, IL-6, and CRP in the MMT-DM group compared to the MMT-Placebo group.

Moreover, dextromethorphan decreased the rate of treatment withdrawal [166]. Additionally, the MMT patients with comorbid pain showed elevated IFN-γ and higher rates of continued opioid abuse [167]. Opioid addicts under MMT also maintained enhanced IL-6 release by the Peripheral Blood Leukocytes. In contrast, the increased production of IL-2, IFNγ, and IL-10 after stimulation was normalized by MMT [143].

These data suggest that several immunological alterations accompany opioid addiction and that neuroinflammation plays a crucial role in opioid dependence and relapse. Notably, a different immunological profile occurs during acute and chronic opioid intake and withdrawal periods and in the periphery versus in the CNS. The available data suggest that IL-1β and TNFα have an essential role in the brain in regulating withdrawal behavior, thus prompting relapse. Moreover, the peripheral levels of TNFα and IL-6 may serve as good predictors for MMT outcomes, although more research is needed. Interventions targeting inflammation arise as potential treatments for opioid addiction (Table 8).

## 6. Conclusions

As discussed in the review, several cytokines, acute-phase proteins, and other immune system mediators reflect how substance use produces both peripheral and central inflammatory alterations. No psychiatric biomarkers are available to support the diagnosis and treatment of substance use disorders. The immune system offers dynamism and complexity, which could reflect the different phases of substance abuse. There is currently a lack of knowledge about the precise alterations that occur at each phase. Therefore, new studies should elucidate the complexity of these variations so that they could be used in routine clinical practice. Further translational studies are needed to avoid the biases that are associated with poly-consumption or the existence of dual pathology.

In conclusion, the use of inflammatory biomarkers in addiction disorders is a promising strategy that is able to reflect the complexity of addictive processes.

## 7. Limitations of the Review

Nonetheless, the results included in the present review should be interpreted with caution due to some limitations. The selected articles were Open Access or covered by CSIC or UMH institutions. Therefore, some articles met our selection criteria, but we were unable to access the full version of the article, and these articles were not included as a result. In addition, several of the studies that were reviewed did not take into account differences in gender, age, or associated comorbidities. Finally, there is no specific information on the duration, magnitude, and drug consumption patterns in many experimental paradigms. Thus, future studies taking all of these variables into consideration are necessary in order to extract more precise information.

## Figures and Tables

**Table 1:** Main findings from animal and clinical studies evaluating changes of inflammatory biomarkers after exposure to cannabinoids.

Target	Experimental Design	Sample	Major Finding	Species	Method	References
IL-1β	Sub-chronic THC administration	Peritoneal macrophages stimulated with LPS	↓ IL-1β just after 10-day THC treatment during adolescence or adulthood	BALB/Cj mice	ELISA Real-time PCR	[43]
		Brain (Hypothalamus and hippocampus)				[44]
		Peritoneal macrophages stimulated with LPS	↑ IL-1β in adult animals as a long-term effect after 10-day THC treatment during adolescence	BALB/Cj mice	ELISA Real-time PCR	[43]
		Brain (Hypothalamus and hippocampus)				[44]
	Chronic cannabis use	Saliva	No significant changes in marijuana users	Human	ELISA	[45]
		Serum	↑ IL-1β in patients diagnosed with Cannabis use disorder	Human	ELISA	[46]
		Serum	No significant changes in long-term marijuana users	Human	Multiplex immunoassay	[47]
IL-1α	Chronic cannabis use	Serum	No significant changes in marijuana users	Human	ELISA	[48]
IL-2	Acute THC administration	Splenocytes	↓ IL-2 after an acute or sub-chronic (7 days) THC exposure	Swiss mice	ELISA	[49]
	Sub-chronic THC administration					
	Chronic THC administration		No significant changes after chronic (14 days) THC treatment			
	Chronic cannabis use	Serum	No significant changes in long-term marijuana users	Human	Multiplex immunoassay	[47]
		Blood	↓ IL-2 in marijuana users	Human	ELISA	[50]
IL-6	Chronic cannabis use	Alveolar macrophages stimulated with LPS	↓ IL-6 in marijuana users	Human	ELISA	[51]
		Bronchial epithelial cells	↑ IL-6 in marijuana users	Human	ELISA	[52]
		Serum	↓ IL-6 in marijuana users	Human	ELISA	[53]
		Plasma	↓ IL-6 in marijuana users	Human	ELISA	[54]
		Serum	↓ IL-6 in marijuana users	Human	ELISA	[55]
		Saliva	↑ IL-6 in marijuana users	Human	ELISA	[45]
		Serum	↑ IL-6 in patients diagnosed with cannabis use disorder	Human	ELISA	[46]
		Serum	No significant changes in long-term marijuana users	Human	Multiplex immunoassay	[47]
		Serum	No significant changes in marijuana users	Human	ELISA	[56]
IL-8	Chronic cannabis use	Bronchial lavage samples	No significant changes in marijuana users	Human	ELISA	[57]
			↑ IL-8 in marijuana and tobacco users			
		Bronchial epithelial cells	No significant changes in marijuana users	Human	ELISA	[52]
		Serum	↑ IL-8 in patients diagnosed with cannabis use disorder	Human	ELISA	[46]
		Serum	No significant changes in long-term marijuana users	Human	Multiplex immunoassay	[47]
IL-10	Chronic cannabis use	Bronchial epithelial cells	No significant changes in marijuana users	Human	ELISA	[52]
		Plasma	No significant changes in marijuana users	Human	ELISA	[54]
		Serum	No significant changes in long-term marijuana users	Human	Multiplex immunoassay	[47]
	Sub-chronic THC administration	Peritoneal macrophages stimulated with LPS	↑ IL-10 after 10-day THC treatment during adolescence or adulthood	BALB/Cj mice	ELISA Real-time PCR	[43]
		Brain (Hypothalamus and hippocampus)				[44]
		Peritoneal macrophages stimulated with LPS	↓ IL-10 in adult animals as a long-term effect after 10-day THC treatment during adolescence	BALB/Cj mice	ELISA Real-time PCR	[43]
		Brain (Hypothalamus and hippocampus)				[44]
		Prefrontal cortex	↓ IL-10 in adult animals as a long-term effect after 11-day THC treatment during adolescence	Sprague Dawley rats	ELISA	[58]
IL-12	Chronic cannabis use	Serum	No significant changes in long-term marijuana users	Human	Multiplex immunoassay	[47]
TNF-α	Chronic cannabis use	Alveolar macrophages stimulated with LPS	↓ TNF-α in marijuana users	Human	ELISA	[51]
		Bronchial epithelial cells	No significant changes (1–10 or 21–40 years of cannabis use)	Human	ELISA	[52]
			↑ TNF-α (11–20 years of cannabis use)			
		Plasma	↓ TNF-α in marijuana users	Human	ELISA	[54]
		Serum	↓ TNF-α in marijuana users	Human	ELISA	[48]
		Saliva	No significant changes in marijuana users	Human	ELISA	[45]
		Serum	↑ TNF-α in patients diagnosed with cannabis use disorder	Human	ELISA	[46]
		Serum	No significant changes in long-term marijuana users	Human	Multiplex immunoassay	[47]
	Sub-chronic THC administration	Peritoneal macrophages stimulated with LPS	↓ TNF-α just after 10-day THC treatment during adolescence or adulthood	BALB/Cj mice	ELISA Real-time PCR	[43]
		Brain (Hypothalamus and hippocampus)				[44]
		Peritoneal macrophages stimulated with LPS	↑ TNF-α in adult animals as a long-term effect after 10-day THC treatment during adolescence	BALB/Cj mice	ELISA Real-time PCR	[43]
		Brain (Hypothalamus and hippocampus)				[44]
		Prefrontal cortex	↑ TNF-α in adult animals as a long-term effect after 11-day THC treatment during adolescence	Sprague Dawley rats	ELISA	[58]
TGF- β	Chronic cannabis use	Alveolar macrophages stimulated with LPS	No significant changes in marijuana users	Human	ELISA	[51]
		Blood	No significant changes in marijuana users	Human	ELISA	[50]
			↑ TGF- β in marijuana and MDMA users			
IFN-γ	Subchronic THC administration	Splenocytes	↓ IFN-γ after subchronic (7 days) THC treatment	Swiss mice	ELISA	[49]
	Chronic THC administration		↓ IFN-γ after chronic (14 days) THC treatment			
	Chronic cannabis use	Serum	No significant changes in patients diagnosed with cannabis use disorder	Human	ELISA	[46]
		Serum	No significant changes in long-term marijuana users	Human	Multiplex immunoassay	[47]
TLR	Chronic cannabis use	Bronchial epithelial cells	↑ TLR2, TLR5, TLR6, TLR9 in marijuana users	Human	ELISA	[52]
CCL11		Plasma	↑ CCL11 in current cannabis users	Human	ELISA	[59]
COX-2	Sub-chronic THC administration	Prefrontal cortex	↑ COX-2 in adult animals as a long-term effect after 11-day THC treatment during adolescence	Sprague Dawley rats	ELISA	[58]
CRP	Acute cannabis use	Serum	No significant changes in marijuana users after recent consumption (after adjusting for covariables)	Human	Nephelometry-based high throughput assay	[60]
		Serum	No significant changes in marijuana users after recent consumption (after adjusting for covariables)	Human	Highly sensitive CRP assay	[61]
		Serum/Plasma	No significant changes in marijuana users after recent consumption (after adjusting for covariables)	Human	Cardiac C-reactive Protein (Latex) Sensitive immunoturbidimetric assay	[62]
	Chronic cannabis use	Plasma	No significant changes in marijuana users	Human	Turbidimetric assay	[54]
		Serum	↑ prevalence of <0.5 mg/dl CRP levels in marijuana users	Human	Latex-enhanced nephelometry	[63]
			↓ CRP in past marijuana users			
			No significant changes in current marijuana users			
		Serum	↓ CRP in current marijuana users	Human	Latex-enhanced nephelometry	[64]
			No significant changes in past marijuana users			
		Serum	No significant changes in marijuana users	Human	Immunoturbidimetric assay	[65]
		Plasma	No significant changes in marijuana users	Human	Highly sensitive CRP assay	[66]
		Whole-blood spots	↑ CRP in marijuana users	Human	Biotin-streptavidin based immunofluorometric system	[67]
		Serum	↑ CRP in long-term marijuana users (associated with ↑ TSPO)	Human	High-sensitivity enzyme-linked immunosorbent assay	[47]
		Serum	↑ CRP in marijuana users (associated with ↑ CVD)	Human	ELISA	[68]
		Serum	↑ CRP in marijuana users (associated with ↑ CVD)	Human	ELISA	[56]

“↑” refers to an increase and “↓” to a decrease in the target of interest.

**Table 2:** Alcohol use disorder: Acute Drinking.

Target	Experimental Design	Sample	Major Finding	Species	Method	References
Mir-223	Acute Drinking	serum	↓neutrophils of alcoholics with recent excessive drinking	Human	Real-time PCR	[83]
Endotoxin			↑ in acute binge drinking in healthy individuals		Endotoxin Kit	[78]
16 rDNA (Bacterial DNA)			↑ in acute binge drinking in healthy individuals		Real-time PCR	
IL-8			↑ in acute binge drinking in healthy individuals		ELISA	[82]
LPS		Plasma	↑ levels in plasma	Wistar Rats	Limulus Amebocyte lysate kit	[79]
Extravesicles miRNA			↓ levels of mir-146a-5p, mir-21-5p, mir-182-5p in females adolescent humans and mice.	Human/C57/BL6 mice	Real-time PCR	[87]
PBMC		Whole Blood	Circulating peripheral blood mononuclear cells ↑ after 20 min of binge drinking and ↓ between 2 and 5 h after ingestion.	Human	Flow Cytometry	[84]
Neutrophils			↑ in alcoholics with recent excessive drinking			[83]
Bacterial composition		Intestinal	↑ Ratio E. Coli/lactobacilli	Wistar Rats	Cell Cultured	[79]

“↑” refers to an increase and “↓” to a decrease in the target of interest.

**Table 3:** Alcohol use disorder: Chronic drinking.

Target	Experimental Design	Sample	Major Finding	Species	Method	References
Magnesium	Chronic Drinking	Serum	↓ magnesium level in heavy drinking AUD patients who exhibited mild liver injury	Human	N.A.	[90]
IgAs against acetaldehyde			Correlation with abstainers, moderate drinkers (1 to 40 g/day), and heavy drinkers (40 to 540 g/day)		ELISA	[92]
Endotoxin			The endotoxemia preceded steatohepatitis	Sprague-Dawley rats	Endotoxin Kit	[96]
C-reactive protein (CRP), IL-6 IL-10		Serum (postmortem)	↑ levels in serum femoral blood obtained from postmortem alcoholic ketoacidosis	Human	ELISA	[95]
IL1β, IL6, IL12, and TNFα (DC)		Whole blood	↑ liberation from peripheral dendritic cells (DC) in patients without liver disease		Flow Cytometry	[94]
TNF-α, IL-1β, NF-κB		Brain Tissue	↑ in hypothalamus	Wistar rats	ELISA	[102]
mRNA Toll-Like Receptors TLR2, TLR3, TLR4			Measure just after ethanol consumption or 24 h. Nucleus accumbens: ↑TLR3 24 h after. And ↓ in the amygdala. TLR4 ↓ in amygdala 24 h after.	C57BL/6 mice	Real-time PCR	[27]
mRNA expression IL-1β and CXCL10			Measure just after ethanol consumption or 24 h. ↑IL-1β in the amygdala and nucleus accumbens just after. CXCL10 ↑ 24 after			
Microglial activation			↑ microglial activation in the cortex		Immunohistochemistry	[29]
mRNA Caspase-1			↑ expression in cortex		Real-time PCR	
NLRP3			↑ protein levels		Western Blot	
Leukocyte			↑ numbers of leukocyte		Flow Cytometry	
IL-1 β, IL-18, IFN-γ, IL-33			↑ level in the whole brain		ELISA	
mRNA CXCL2 (MIP2-α), CX3CL1 (Fractalkine)			↑ mRNA levels		Real-time PCR	
mRNA CCL4 (MIP-1β)			↓ mRNA levels			
TNFα, MCP1, and IL-1β			↑ in cerebellum		ELISA	[98]
miR-155			↑ in cerebellum		Real-time PCR	
mRNA IL-1β, IL-6, MCP-1			↑ whole brain expression mRNA levels			[101]
MCP-1		Brain Tissue (Postmortem)	↑ Level in VTA, SN, Hippocampus, and Amygdala	Human	ELISA	[100]
Iba-1+, Glut5			↑ microglial, iba-1+ and glut5+ cells, in cingulated cortex		Immunohistochemistry	
Glut5			↑ microglial, Glut5 positive cells, in midbrain and VTA			
mRNA TLR7			↑ expression in hippocampus		Real-time PCR	[28]
TLR7, HMGB1, CD11b			↑ in hippocampus		Western Blot	
miR181b-3p		Liver	↓ miRNA level in liver	C57BL/6J	RNA Next-Generation Sequencing	[97]
oral sugar test		Urine	↑ gut permeability before the development of alcoholic liver disease	Sprague-Dawley rats	Gas chromatography	[96]
TSPO activity		Neuroimaging	↑ Binding with F-DPA-714 during and 7 months after exposure	Non-human primates	PET	[104]
			↑ Binding with (^11^C)PBR28	Wistar rats		[103]
			↓ Binding with (^11^C)PBR28 in alcoholic patients admitted in rehabilitation	Human		[103] [105] [106]

“↑” refers to an increase and “↓” to a decrease in the target of interest.

**Table 4:** Alcohol Use Disorder: Withdrawal.

Target	Experimental Design	Sample	Major Finding	Species	Method	References
IL-6, IL-8, IL-10	Withdrawal	Serum	↓ levels after a few days of abstinence in patients with an alcohol withdrawal syndrome	Human	ELISA	[82]
Advanced glycation end products (AGE)			↑ In patients with at least one month of abstinence compared to control.		Spectrophotometric	[112]
IL-1RA, Il-8, Il-6			↑ Alcoholic patients without liver disease one month of detoxification, compared with control.		ELISA	[111]
Clara cell secretory protein (CC16)			↓ Alcoholic patients without liver disease one month of detoxification			
CCL18		Subcutaneous Adipocyte tissue	↑ after one week of withdrawal in ALD patients		Real-time PCR	[108]

“↑” refers to an increase and “↓” to a decrease in the target of interest.

**Table 5:** Table: Alcoholic Liver Disease.

Target	Experimental Design	Sample	Major Finding	Species	Method	References
sST2	Alcoholic Liver Disease (ALD)	Plasma	It was positively correlated with Maddrey discriminant function (MDF), Child–Pugh scale, IL-6 Il1B, and ALD severity.	Human	ELISA	[117]
High-sensitivity C-reactive protein (hsCRP)			It was correlated with the liver dysfunction marker and hepatic venous pressure. On the contrary, it had a negative correlation with survivability.			[115]
YKL40			↑ hit the severity of fibrosis and hepatic inflammation			[119]
IL-1β, IL-6, and TNF-α		Serum	Binge and escalating alcohol exposure ↑ serum levels.	Wistar rats		[116]
IL-10			Binge and escalating alcohol exposure ↑ at the end of 4 and 8 weeks but ↓ after that and was significantly decreased at 12 and 16 weeks.			
HSP70, TNFα			Depending on the severity of the Alcoholic Fatty Liver Disease	Human		[114]
IL-17A, IL-1beta, IL-6			↑ IL-6 highest diagnostic and prognostic biomarker to the fatal ALD course			[120]
Th17/Treg		Whole Blood	↑ Th17 and ↓Treg frequencies were observed in non-survivors		Flow Cytometry	
mRNA expression of Toll-Like Receptors		Liver	Upregulated TLR1, 2, 4, 6, 7, 8, and 9 (TLR10, TLR11 Not tested)	C57Bl6/J	Real-time PCR	[123]
Microglia		Brain Tissue (Postmortem)	↑ in the subventricular zone (SVZ) in an alcoholic with cirrhosis and hepatic encephalopathy	Human	Immunohistochemistry	[124]
IL-6			↑ in superior frontal gyrus (SFG), the precentral gyrus (PCG) in an alcoholic with cirrhosis and hepatic encephalopathy		ELISA	
		Acute Alcoholic Hepatitis				
sCD163		Plasma	Positive correlation with severity and mortality of Acute Alcoholic Hepatitis (AAH)	Human	ELISA	[125]
IgM, IgA, IgG against *p. gingivalis* 33277 and w83			Progression and severity of Acute Alcoholic Hepatitis (AAH)			[126]
IFN-γ		Serum	Negative association with low levels of IFN-γ at admission and long-term mortality in patients with Acute Alcoholic Hepatitis (AAH)			[122]
Il-6			Patients with IL-6 ≥ 38.66 pg/mL had significantly decreased mean survival than those with lower levels in Acute Alcoholic Hepatitis.			[121]
CD163		Liver	↑ in patients with Acute Alcoholic Hepatitis (AAH)			[125]

“↑” refers to an increase and “↓” to a decrease in the target of interest.

**Table 6:** Opioid use disorder: Acute Opioid Administration.

Target	Experimental Design	Sample	Major Finding	Specie	Method	References
IL-1β, IL-10	Acute Opioid Administration	Serum	↑IL-10	Sprague Dawley rats	ELISA	[130]
CRP, IFN-γ, CXCL9, CCL11, CCL12, CCL25, CCL17, CCL4, CCR4, CX3CL1, IL-10		Brain Tissue	↑ CRP, IFN-γ, CXCL9, CCL11, CCL12, CCL25, CCL17, CCL4, CCR4, IL-10 ↓ CX3CL1 Increased levels of IL-10 in Nacc are correlated with reduced risk of opioid-dependence		Real-time PCR	
CCL5, IL-1β, TNFα			↑ IL-1β in cortex, hippocampus, and striatum ↑ TNFα	Sprague Dawley rats	ELISA	[132]
IL-6, IL-1β		Serum	No change	Sprague Dawley rats	ELISA	[131]

“↑” refers to an increase and “↓” to a decrease in the target of interest.

**Table 7:** Opioid Use Disorder: Chronic Opioid Exposure.

Target	Experimental Design	Sample	Major Finding	Species	Method	References
IL-6, IL-1β	Chronic opioid exposure	Serum	↑ IL-6, IL-1β after 6 days of morphine administration.	Sprague Dawley rats	ELISA	[131]
IL-6, IL-1β		Brain Tissue	↑ IL-6, IL-1β mRNA in NAcc and mPFC, after 6 days of morphine administration.		Real-time PCR	
IL-6, IL-1β			↑ IL-6, IL-1β level in NAcc and mPFC, after 6 days of morphine administration.		ELISA	
IL6, TNFα, NF-kB, iNOS			↑ NF-kB, iNOS, TNFα, IL-6, after 8 weeks of tramadol administration.	Albino rats	Real-time PCR, WB	[134]
IL6, TNFα		Serum	↑ IL-6, TNFα, after 8 weeks of tramadol administration.		Real-time PCR	
CRP, TNF-α, IL-17A			↑ IL-6, TNFα, after 14 days of 50 mg/kg treatment of tramadol or tapentadol. ↓ IL-17A only at 50mg/kg of tapentadol	Wistar rats	ELISA	[135]
IL-1β, TNF, IL-6, CXCL1			↑ IL-1β, IL-6, TNFα, CXCL1 in a model of perinatal methadone exposure	Sprague Dawley rats	Multiplex Electrochemiluminescent Immunoassay (MECI)	[136]
IL-1β, TNF, IL-6		Peripheral blood mononuclear cells	↑ Basal TNFα ↑ TNFα, IL-1β after LPS stimulation, in a model of perinatal methadone exposure.		MECI	
IL-1β, CXCL1, TNF, IL-6		Brain Tissue	↑ TLR4, MyD88, IL-1 β, CXCL1 in the cortex, in a model of perinatal methadone exposure.		Real-time PCR, MECI	
IL6, TNFα, TLR2, CD11b			↑ IL-6, TNFα, TLR2, CD11b in NAcc after 3 days of morphine administration.	C57BL/6 mice	Real-time PCR	[137]
TLR4			↑ TLR4 mRNA in NAcc, after prolonged remifentanil self-administration.	Sprague Dawley rats	Real-time PCR	[138]
CCL5, IL-1β, TNFα			↑ CCL5 in cortex and striatum ↓ IL-1β in the cortex, after scaling morphine administration for 5 days.	Sprague Dawley rats	ELISA	[132]
CXCL12			↑ CXCL12 in VTA, after 6 days of morphine administration.	Sprague Dawley rats	Real-time PCR, WB	[139]
Adaptive immunity markers		Serum	↑ Total B cells ↑ IgG3, IgG4, IgM ↑ sCD40l, TNFα, TGFα, IL-8, endotoxin. In active heroin injection drug users.	Human	Flow cytometry, ELISA, LAL	[140]
CRP, C3 and C4, IgM, IgA, antioxidant capacity (TAC)			↑ CRP, C3, C4, IgA, TAC in chronic opium smokers.		ELISA, FRAP	[141]
TNFα		Plasma	↑ TNFα in OUD patients.		ELISA	[142]
Immunological parameters		Serum	↑ white blood cell, neutrophil count, and neutrophil percentage were ↓ lymphocyte percentage, and basophils count ↑ inflammatory indexes. In OUD patients.		N.D.	[141]
PBL proliferation, IL-2, IL-4, IL-6, IL-10, IFNγ		Whole blood	↑ Peripheral Blood Leukocytes (PBL) proliferation ↑ Basal IL-6 ↑ IL-10 release after stimulation. In heroin addicts.		ELISA	[143]
IL-1β, IL-15, CD15, CD68, IL-8, IL-10, TNFα, IL-6, COX-2, HSP-70, IRP-150		Brain Tissue (Postmortem)	↑ IL-15, CD68, TNF-a, IL-6; COX-2, HSP-70, ORP-150, in heroin-related deaths.		Immunohistochemistry, WB	[144]
TNFα, IL-8		Plasma	↑ TNFα, IL-8 in heroin-dependent patients. TNFα, IL-8 were correlated to years using heroin		ELISA	[145]
Dysregulated gene expression		Brain Tissue (Postmortem)	OUD patients exhibit an enhanced expression of transcripts related to neuroinflammation. Top activated upstream regulators were identified to be TNFα, IL-1β, NFkB in DLPFC and IL-1β in NAcc.		RNA Next-Generation sequencing	[146]
			↑ Gene module firebrick3 (associated with immune responses) in opioid abuse group.			[147]
			IL4 and PECAM1 are identified as critical genes for opioid addiction.			[148]

“↑” refers to an increase and “↓” to a decrease in the target of interest.

**Table 8:** Opioid Use Disorder: Withdrawal.

Target	Experimental Design	Sample	Major Finding	Species	Method	References
IL-1β, TNFα	Withdrawal	Brain Tissue	↑ IL-1β, TNFα Related with withdrawal behavior in hippocampus	Wistar rats	ELISA	[158]
IL-1β			↑ IL-1β Related with withdrawal behavior in hippocampus	C57BL/6 mice		[159]
TNFα			TNFα is required for withdrawal behavior in Lateral Habenula	C57BL/6J mice	N.A.	[160]
↑ TNF-α, IL-6, IL-1β, IL-10			↑ TNF-α, IL-6, IL-1β, IL-10	Swiss mice	ELISA	[161]
CCL5, IL-1β, TNFα			↑ TNF-α in striatum ↑IL-1β in hippocampus and striatum ↓ IL-1β in cortex ↓ CCL5 in cortex	Sprague Dawley rats		[132]
Differentially expressed genes in neurons, microglia and astrocytes			↑ TNF-α in the three cell types ↑ Shift in astrocytes in CeA	Sprague Dawley rats	Real-time PCR	[157]
IL1-B, IL-6, IL-8, IL-10, TNFα		Plasma	↑ IL-1β, IL-6, IL-8	Human	Flow Cytometry	[163]
IL-6, CRP, TNFα, TGF-1B		Serum	↓ TNF-α, CRP, IL-6, TGF-β1 after MMT		Antibody pair assay system	[164]
TNFα			↑ TNFα at baseline ↓ TNFα after MMT		ELISA	[142]
Plasma TNFα, CRP, IL-6, TGF-1β		Serum, Urine	↓ CRP, TGF-β1 after MMT Higher IL-6 levels were associated with poor MMT outcomes.		Antibody pair assay system	[165]
TNF, IL-1, IL-1ra, IL-6, IL-8, IL-10, IFN-, CCL2, in relation with chronic pain		Serum	↑ IFN-γ ↑ Positive opioid urine screen		Bio-Plex assay	[167]
TNFα, IL-8		Plasma	↓ TNFα, IL-8 in MMT-Dextromethorphan, not in MMT-Placebo		ELISA	[145]
TNFα, IL-8, IL-6, TGFα, CRP			↓ TNFα in MMT-DM compared to MMT-Placebo		Antibody pair assay system	[166]

“↑” refers to an increase and “↓” to a decrease in the target of interest.
